# Superior vena cava replacement combined with venovenous shunt for thymic carcinoma

**DOI:** 10.1016/j.ijscr.2020.05.069

**Published:** 2020-06-06

**Authors:** Soichi Oka, Kenji Ono, Yoshio Arai, Kenta Kajiyama, Katsuma Yoshimatsu, Takehiko Manabe

**Affiliations:** a Thoracic Surgery, Kokura Memorial Hospital, Kitakyushu, Japan; b Cardiovascular Surgery, Kokura Memorial Hospital, Kitakyushu, Japan

**Keywords:** CT, computed tomography, SVC, superior vena cava, RBCV, right brachiocephalic vein, LBCV, left brachiocephalic vein, VATS, video-assisted thoracic surgery, PTFE, polytetrafluoroethylene, Superior vena cava, Thymic carcinoma, Surgery

## Abstract

**Introduction:**

Advanced-stage thymic malignancies are a heterogeneous group of mediastinal tumors that include thymoma and thymic carcinoma infiltrating the surrounding thoracic structures. When the tumor infiltrates the superior vena cava (SVC), radical resection can be selectively achieved via en bloc SVC resection and its prosthetic conduit replacement. We herein report a case of SVC replacement for thymic carcinoma en bloc radical resection.

**Case presentation:**

A 75-year-old Japanese man presented at our hospital due to progressive dyspnea and edema of his face and upper extremities. CT showed a 55 × 40 × 38-mm tumor located at the anterior mediastinum lesion. This tumor had invaded the superior vena cava and both brachiocephalic veins. We performed surgical resection for the thymic carcinoma located at the mediastinum that invaded the superior vena cava and both brachiocephalic veins. The surgery was performed through a full median sternotomy and transmanubrial approach without using an artificial heart and lung. The tumor involved the SVC, right brachiocephalic vein (RBCV) and left brachiocephalic vein (LBCV). We performed SVC replacement for thymic carcinoma en bloc radical resection.

**Discussion:**

This report has two important implications. First, a venovenous shunt (VVS) from the distal LBCV to the right auricle was very useful and safe before performing an SVC complete clamp. The second implication of our study was that using a PTFE with a large inner diameter may prevent thrombus occlusion.

**Conclusions:**

We experienced SVC replacement for thymic carcinoma en bloc radical resection. We were able to safely performed this surgery using our usual approach.

## Highlights

• SVC replacement for lung cancer or thymoma is infrequently performed and technically challenging.
• We experienced SVC replacement for thymic carcinoma en bloc radical resection.
• We were able to safely performed this surgery using our usual approach.

## 1 Introduction

Advanced-stage thymic malignancies are a heterogeneous group of mediastinal tumors that include thymoma and thymic carcinoma infiltrating the surrounding thoracic structures. Surgery is indicated in select stage III and sometimes certain stage IV cases as part of a multimodality therapeutic approach. When the tumor infiltrates the superior vena cava (SVC), radical resection can be selectively achieved via en bloc SVC resection and its prosthetic conduit replacement [1].

Locally advanced lung or primary mediastinal malignancies are the most common causes of SVC syndrome, accounting for 60 % of cases [2]. However, improvements in surgical techniques and neoadjuvant therapy have made it possible to eliminate clinical symptoms as well as achieve favorable immediate and long-term outcomes [3].

We herein report our experience in SVC replacement for thymic carcinoma en bloc radical resection. This work has been reported in line with the SCARE criteria [13].

## 2 Case presentation

**Fig. 1:**
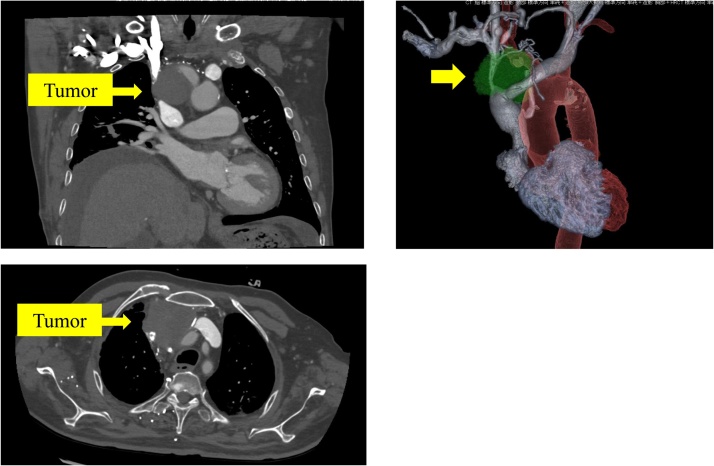
Chest computed tomography (CT) scan showing a 55 × 40 × 38-mm tumor located at the mediastinal lesion. This tumor had invaded the superior vena cava and both brachiocephalic veins.

A 75-year-old Japanese man presented at our hospital due to progressive dyspnea and edema of his face and upper extremities. His medical history included chronic renal failure with peritoneal dialysis. On a physical examination, there was facial swelling and venous dilation of the neck and the chest wall. Other physical examination results were normal. Chest computed tomography (CT) showed a 55 × 40 × 38-mm tumor located at the anterior mediastinum. This tumor involved the SVC, both brachiocephalic veins, the pericardium and the right upper lung lobe (Fig. 1). His blood examination results revealed an increased C-reactive protein level without leukocytosis. The findings of serologic tumor markers were negative.

We performed a video-assisted thoracic surgery (VATS) biopsy from the right thoracic cavity to make a definitive diagnosis. The tumor was poorly differentiated thymic carcinoma. There was no significant distant metastasis. Therefore, we performed surgical resection for this thymic carcinoma located at the mediastinum with invasion of the SVC and both brachiocephalic veins.

**Fig. 2:**
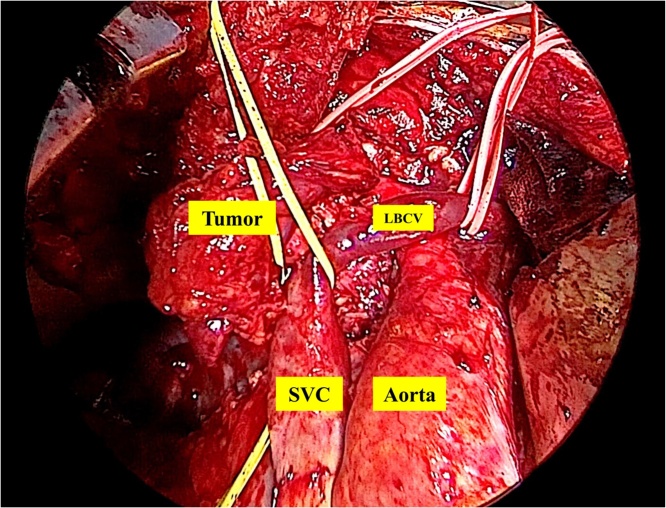
Operation findings showed that the tumor was involved with the SVC, right brachiocephalic vein (RBCV) and left brachiocephalic vein (LBCV).

**Fig. 3:**
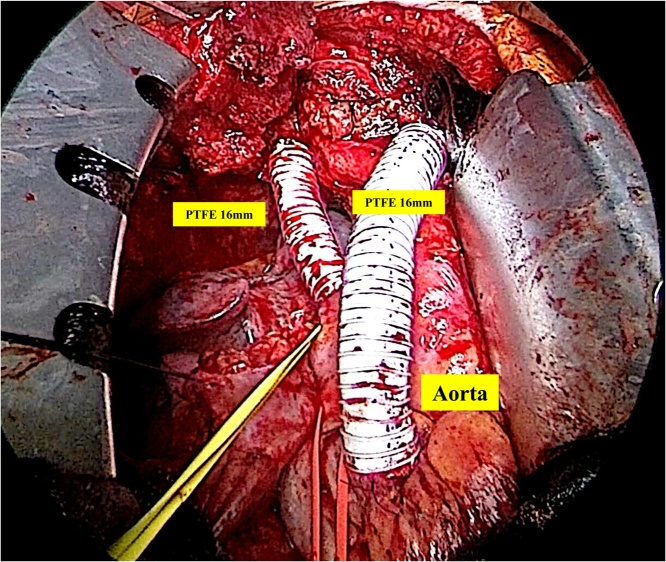
Operation findings showed that we achieved SVC replacement with PTFE conduits.

SVC replacement using an expanded polytetrafluoroethylene (PTFE) (16 mm inside diameter) was chosen. The surgery was performed through a full median sternotomy and transmanubrial approach without using an artificial heart and lung. Pericardiotomy was performed to clarify the tumor involvement at the epicardium. Tumor invasion was not found in the pericardium. The tumor involved the SVC, right brachiocephalic vein (RBCV) and left brachiocephalic vein (LBCV) (Fig. 2). First, we performed a venovenous shunt between the distal LBCV and right auricle. We checked the pressure of both internal jugular veins throughout the operation. Next, the RBCV was anastomosed to the distal end of an expanded PTFE graft (16 mm inside diameter). The proximal end of the graft was anastomosed to the root of the SVC. The LBCV was anastomosed to the distal end of an expanded PTFE graft (16 mm inside diameter). The proximal end of the graft was anastomosed to the right auricle (Fig. 3). The tumor had invaded the right upper lobe of the lung. Finally, we performed en bloc resection of the tumor with part of the right upper lobe of the lung. The operative time was 6 h 15 min, and the amount of intraoperative bleeding was 1360 mL.

Pathologically, complete resection was achieved. The pathological diagnosis was stage IIIA poorly differentiated thymic carcinoma (p-T3N0M0 stage IIIA). The patient was discharged from our hospital 22 days after surgery. This surgery was successful, with no postoperative complications.

## 3 Discussion

SVC replacement for lung cancer or thymoma is infrequently performed and technically challenging, especially in a facility lacking relevant experience. One of the major obstacles is how to shorten the clamping time of the SVC in order to reduce neurological complications. Although SVC replacement has been well documented, there are no data showing which method is best for reducing the SVC clamping time [[3], [4], [5],^9^].

We experienced a case of SVC replacement for thymic carcinoma en bloc radical resection. This report has two important implications. First, a venovenous shunt (VVS) from the distal LBCV to the right auricle was very useful and safe before performing an SVC complete clamp, and we constantly monitored the internal jugular vein pressure during the operation. Therefore, we were able to safely perform SVC clamping and replacement by monitoring the cerebral intravascular pressure at all times. The application of a VVS, i.e. a vena cava bypass or caval shunt, as reported by Yoshimura [6], has been reported a few case reports [3,7]. However, the indications and details for performing VVS have not been clarified [8]. Dai et al. reported that the median SVC clamping time was 75 min, which was much longer than the time reported in the literature. Every effort should be made to reduce the clamping time as much as possible, especially in patients with an incompletely obstructed SVC system [8]. Complete vessel clamping is usually tolerated up to 45−60 min when appropriate pharmacological support is provided [1,10]. Due to a lack of experience and concern about intraoperative emergencies, Dai et al. often instituted a temporary VVS before surgery and removed it afterwards in order to maintain stable hemodynamics during SVC clamping [8]. Based on their experiences with VVSs, they suggested instituting an external VVS preoperatively when a center has just begun to perform SVC replacement procedures, especially for patients without chronic SVC system obstruction. In addition, they felt that preoperative planned external VVS was preferred, especially when two veins were being replaced, as intraoperative catheter insertion for the internal VVS would be much more difficult, especially when performed at the relatively distal part of the SVC. Furthermore, they found that performing an intraoperative internal VVS was a viable alternative at experienced centers, especially when emergencies occur [8].

The second implication of our study was that using a PTFE with a large inner diameter may prevent thrombus occlusion. Conduit replacement of the SVC is usually indicated for infiltration of more than 30 % of the vessel circumference. Some authors have reported that conduit reconstruction is indicated for infiltration exceeding 50 %. Maurizi et al. reported that patch reconstruction after resection of more than 30 % of the SVC circumference might lead to a higher risk of kinking, thrombosis and occlusion [1]. In the present case, the SVC infiltration area was more than 90 %. We therefore performed SVC reconstruction using the PTEF conduit. Some previous reports have described cases of SVC segment resection with innominate vein-right atrium double bypass [10,11]. Maurizi et al. recommended that this kind of reconstruction be avoided, as the blood flow through the bypass is too low and creates a high risk of thrombosis [1]. Therefore, in our case, SVC replacement using a PTFE (16 mm inside diameter) was chosen. This patient has had no obstruction due to thrombus in the PTFE in the four months since the operation with anticoagulant therapy.

SVC replacement combined with VVS is technically feasible and safe. However, the best strategy for SVC replacement remains controversial [1,12]. We need to accumulate more cases in order to establish safe and effective procedures for SVC replacement.

## 4 Conclusion

We experienced SVC replacement for thymic carcinoma en bloc radical resection. We were able to safely perform this surgery using our usual approach. We need to accumulate more cases in order to establish safe and effective procedures for SVC replacement.

## Declaration of Competing Interest

We have no conflicts of interest.

## Funding

We have no sources of funding for our research.

## Ethical approval

We got ethical approval from ethical committee of Kokura memorial hospital, Japan.

## Consent

We had informed consent from this patient for writing this paper.

## Author contribution

Soichi Oka; study design, writing. Kenji Ono; study design, other. Yoshio Arai; other. Kenta Kajiyama; other. Katsuma Yoshimatsu; other. Takehiko Manabe; other.

## Registration of research studies

My research registry number is 1565.

## Guarantor

Soichi Oka and Kenji Ono.

## Provenance and peer review

Not commissioned, externally peer-reviewed.
